# Sustained Ventricular Tachycardia in a Patient with Thalassemia Major

**DOI:** 10.1111/anec.12085

**Published:** 2013-09-09

**Authors:** Nermin Bayar, Şakir Arslan, Zehra Erkal, Selçuk Küçükseymen

**Affiliations:** ^1^ Antalya Education And Research Hospital Cardiology Department Antalya Turkey

**Keywords:** electrophysiology–ventricular tachycardia, clinical, implantable devices–ventricular tachycardia/fibrillation, clinical

Although improvements in the treatment of thalassemia with transfusion and chelation therapy have been achieved, iron overload because of repeated red blood cell transfusions continue to cause organ dysfunction. The prognosis is determined by the degree of involvement of heart disease, mortality in these patients is mainly because of heart failure.^1^ In this case, cardiac hemosiderosis due to thalassemia and admitted to our Emergency Room with ventricular tachycardia with hemodynamic disrupts, then have implantable cardioverter defibrillator (ICD), 30‐year‐old male patient is presented. This is the first report among the thalassemia patients, presented with ventricular tachycardia based on literature search.

## CASE REPORT

**Figure 1:**
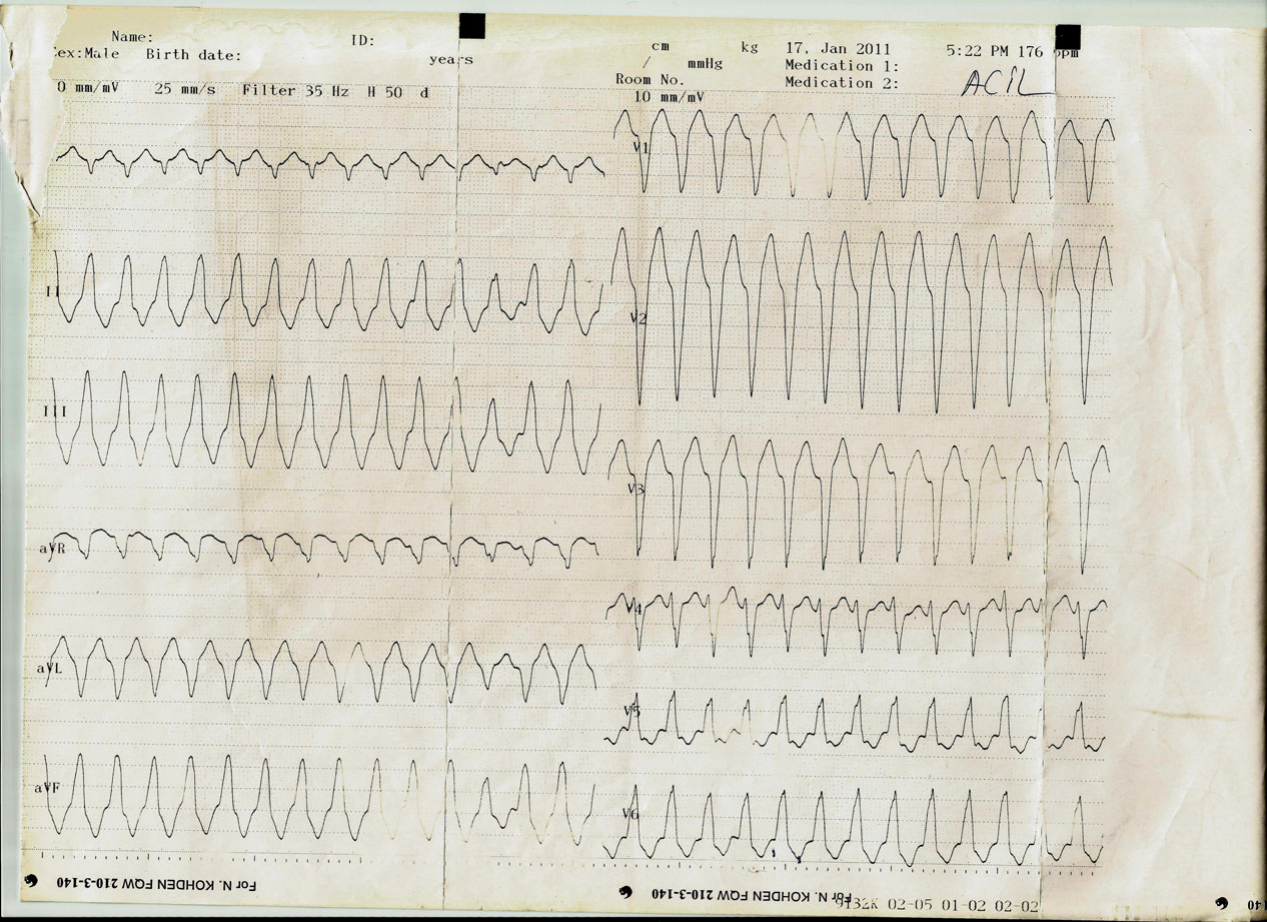
ECG on admission to emergency department.

**Figure 2:**
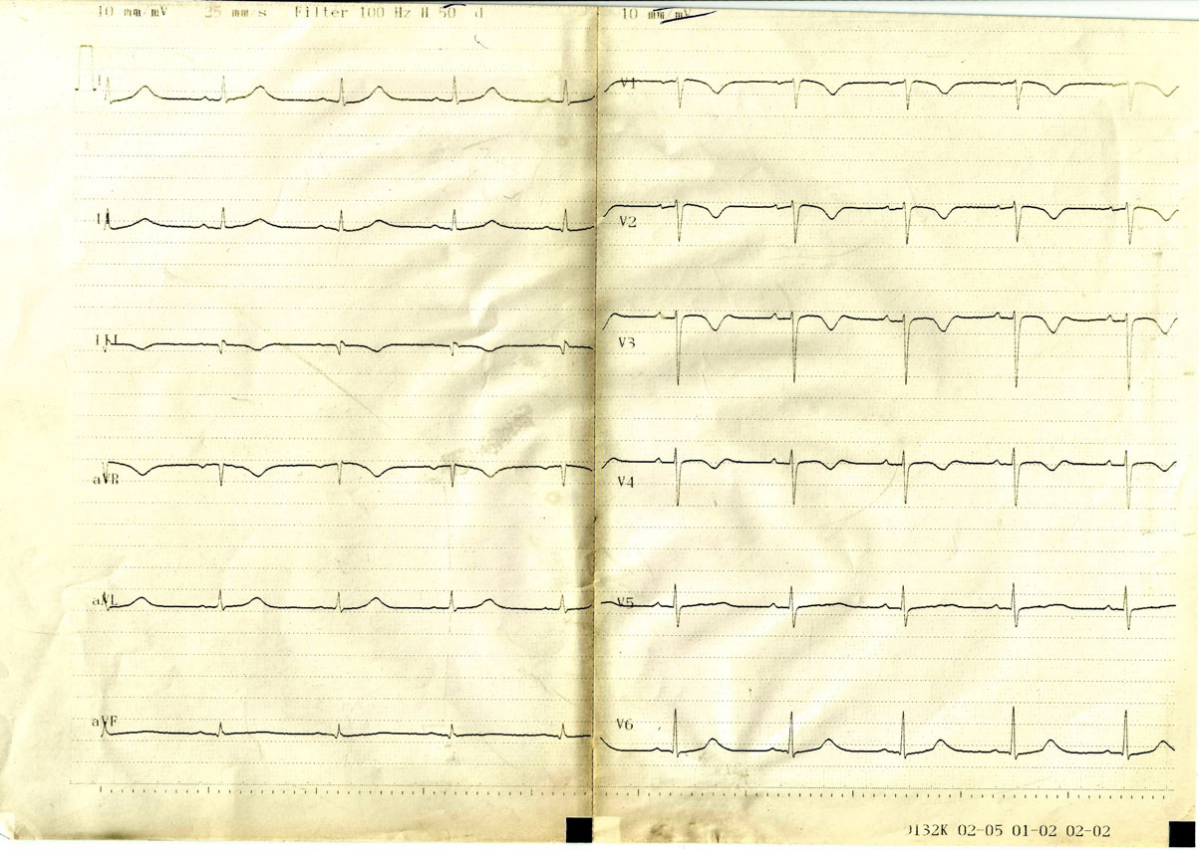
ECG after being defibrillated.

**Figure 3:**
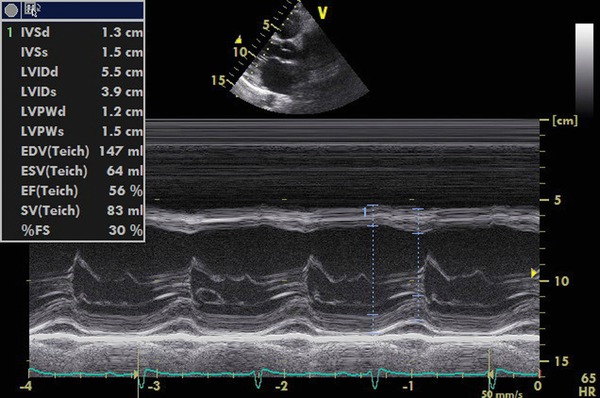
M‐mode imaging, the left ventricular dimensions and ejection fraction were normal.

**Figure 4:**
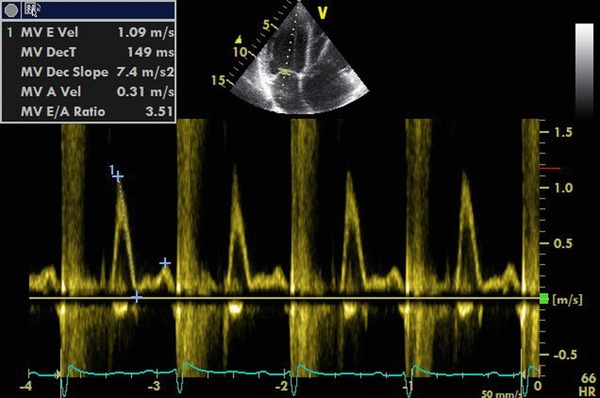
Mitral E/A ratio were above 2.

**Figure 5:**
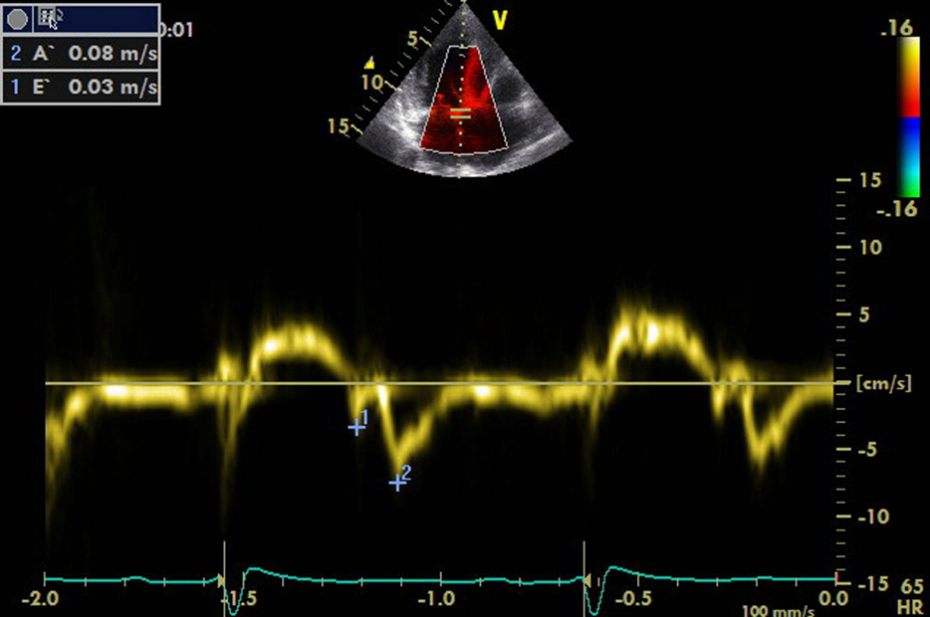
Mitral E′ value is below 8 ms and consistent with restrictive type diastolic dysfunction.

**Figure 6:**
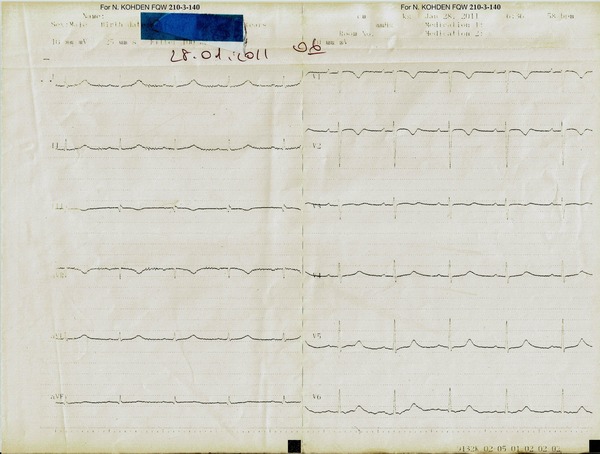
Patient's ECG, has viewed repolarization changes during follow‐up.

Thirty‐year‐old male patient, who followed with a diagnosis of β‐thalassemia major, was admitted to the emergency room with palpitations and chest tightness before half an hour. There was a history of blood cell transfusion for thalassemia since the age, 8 years old began to deferoxamine, 10‐year‐old splenectomy was performed and before 4 months was started deferiprone in addition to deferoxamine. There was increase in dyspnea and palpitation in the past few months. During the application, blood pressure was 90/50 mm Hg, the pulse 176/minute, an electrocardiogram showed wide‐complex tachycardia (Fig. 1). The patient initially received intravenous amiodarone (300 mg/30 min) and transfered to coronary intensive‐care unit. 15th minute of infusion, the patient became hypotensive and hemodynamically unstable, lost consciousness, pulseless ventricular tachycardia (VT) were defibrillated with 200 joules and sinus rhythm was achieved (Fig. 2). Then installation of intravenous amiodarone was completed and the maintenance of oral treatment was passed, metoprolol 50 mg/day and ramipril 2.5 mg/day was administered. Transthoracic echocardiography revealed normal left ventricular systolic function, left ventricular hypertrophy and diastolic *dysfunction* have a *restrictive filling* pattern (Figs. 3, 4, and 5). The hemogram reveals profound anemia with a hemoglobin level of 8.4 g/dL, diluted ferritin was 1300 ng/mL, thyroid hormones, electrolyte levels and cardiac enzymes were normal. Erythrocyte suspension were given the suggestion of hematology clinic, chelation therapy was continued. The patient was taken to control ventricular arrhythmias with amiodarone, ICD implantation was performed for the purpose of secondary prevention. Patient's ECG, has viewed repolarization changes during follow‐up (Fig. 6). Follow‐up of patient without complication and was discharged with metoprolol and amiodarone therapy.

## DISCUSSION

β‐thalassemia is an inherited blood disease caused by mutations leading to reduction or absence of the β‐globin chain production.^2^ Clinical features of β‐thalassemia include haemolytic and hyperchromic anemia, ineffective erythropoiesis that causes profound anemia. Conventional therapeutic methods for β‐thalassemia are regular transfusion, chelating therapy and induction of fetal hemoglobin synthesis, iron overload results both transfusional hemosiderosis and excessive gastrointestinal iron absorption, so the characteristic features of advanced iron overload are failure of vital organs such as liver and heart in addition to endocrine dysfunctions.

Cardiac complications of thalassemia are heart failure, arrhythmias, pericarditis, and including pulmonary hypertension and right heart failure, more intermediate thalassemia have been reported. Cardiac iron overload in patients with thalassemia is particularly important, 71% of deaths in these patients because of cardiac origin, and deaths due to heart failure or arrhythmias associated with sudden death.^3^

As the age of the patients and iron loads, has shown a progressive arrhythmias. 22–65% of cardiac hemosiderosis patients were analyzed ECG abnormalities. PR prolongation, T‐wave abnormalities, low QRS voltage, intraventricular conduction defect, and atrial fibrillation have been reported in patients with thalassemia.^4^ Twenty‐four hour Holter monitoring in patients with iron overload, atrial and ventricular extrasystoles and intermittent episodes of VT were determined.^5^ Persistent atrial standstill and idioventricular rhythm observed in the literature, cases have been reported in thalassemia intermedia.^6^

Kardelen et al. detected that heart rate variability in patients with thalassemia major was seen to be significantly lower than the control group, in the early period the patients with cardiac autonomic

neuropathy have been reported.^7^ In another study, β‐thalassemia major patients, secondary to increased sympathetic activation and atrial dilatation, as a result of intra‐atrial conduction delay, prolonged P‐wave dispersion has been reported.^8^

Ventricular arrhythmias, atrial arrhythmias in patients with thalassemia are rare and usually more resistant to antiarrhythmic therapy. Isma'eel et al., the incidence of ventricular late potentials on signal averaged ECG in patients with thalassemia is more than the normal individuals were determined.^9^ Kocharian et al., QT interval and QT dispersion which predictors of sudden cardiac death were investigated and in patients with thalassemia major is longer than in normal individuals reported.^10^ These studies in patients with thalassemia, show that the tendency to ventricular arrhythmias increased. Our patient had sustained VT with hemodynamic instability and an ICD implantation was decided for secondary prevention.

## CONCLUSION

Thalassemia major patients are predisposed to arrhythmias, thus early recognition of cardiac involvement is important for life quality and expectancy of patients.
